# Evolutionary Mechanisms of Varying Chromosome Numbers in the Radiation of *Erebia* Butterflies

**DOI:** 10.3390/genes9030166

**Published:** 2018-03-16

**Authors:** Kay Lucek

**Affiliations:** Department of Environmental Sciences, University of Basel, Schönbeinstrasse 6, 4056 Basel, Switzerland; kay.lucek@unibas.ch

**Keywords:** Lepidoptera, *Erebia*, adaptive radiation, chromosomal rearrangement, intrinsic barriers

## Abstract

The evolution of intrinsic barriers to gene flow is a crucial step in the process of speciation. Chromosomal changes caused by fusion and fission events are one such barrier and are common in several groups of Lepidoptera. However, it remains unclear if and how chromosomal changes have contributed to speciation in this group. I tested for a phylogenetic signal of varying chromosome numbers in *Erebia* butterflies by combining existing sequence data with karyological information. I also compared different models of trait evolution in order to infer the underlying evolutionary mechanisms. Overall, I found significant phylogenetic signals that are consistent with non-neutral trait evolution only when parts of the mitochondrial genome were included, suggesting cytonuclear discordances. The adaptive evolutionary model tested in this study consistently outperformed the neutral model of trait evolution. Taken together, these results suggest that, unlike other Lepidoptera groups, changes in chromosome numbers may have played a role in the diversification of *Erebia* butterflies.

## 1. Introduction

The process of speciation can be seen as a continuum, ranging from initially undifferentiated populations to ultimately genetically distinct and reproductively isolated species [1,2,3]. Early stages along this continuum are often ephemeral, as potential maladaptive gene flow is mainly prevented by extrinsic factors, and interspecific gene flow, e.g., upon secondary contact, is still possible [2,3,4]. The evolution of intrinsic barriers that shield part of the genome from recombination and prevent the breakup of co-adapted genomic regions is thus vital for the progression of speciation [5,6]. Intrinsic barriers may not only stabilize initial differentiation [3,7,8], but also promote the subsequent coexistence of closely related species [9,10]. Theory supports the idea that chromosomal rearrangements, such as translocations, inversions and the change in chromosome numbers through fusion and fission of existing chromosomes, can act as intrinsic barriers [11,12,13]. Changes in chromosome numbers through fusion and fission may, in particular, result in reproductive isolation, and thus promote speciation. This is because complex and unstable meiotic chains can form in hybrids between species with different chromosome numbers, leading to meiotic nondisjunction and sterility [14]. Hybrid sterility may, however, take a long time to establish. This is indicated by often widespread hybridization between closely related species or chromosomal races in the wild [15,16], especially in butterflies [17]. Recent genomic data further suggests that chromosomal barriers may be more porous than formerly thought [18]. Differences in chromosome numbers nevertheless often reduce gene flow and thus provide some initial degree of isolation [19]. The extent to which such differences contribute to speciation remains yet unknown [12,13,20,21].

Speciation through fusion and fission of chromosomes has, for example, been suggested to drive diversification in some groups of mammals [13,20] or Lepidoptera [21,22,23]. Chromosome numbers are often highly conserved among many Lepidoptera genera and families with a haploid chromosome number (*n*) of ~31 [22]. However, several groups of butterflies, such as *Agrodiaetus* [21] or *Lysandra* [24], exhibit high karyotypic diversity [22]. The *Agrodiaetus* group (*n* range: 10–134) has become a model system for studying the evolutionary interplay between chromosome numbers, speciation and the coexistence of species [21,23]. In contrast, the evolutionary mechanisms remain unclear for most other Lepidopteran groups, including *Erebia*. *Erebia* is a genus of Palearctic butterflies consisting of more than one hundred described species that differ phenotypically, ecologically and karyotypically from each other [22,25,26]. Phylogenetic inferences suggest that the radiation of European *Erebia* emerged over the last ~15 million years [26]. However, among European *Erebia*, the *E. tyndarus* group is much younger (0.15–1 million years; [26,27,28]). The process of speciation is not complete in the *E. tyndarus* group, as interspecific gene flow is still possible [17,26,28]. Similar to *Agrodiaetus*, speciation in *Erebia* is suggested to be driven by changes in chromosome numbers (*n* range: 7–51), associated with allopatric phases followed by secondary contact [26,29]. The high karyotypic diversity in *E. tyndarus* (*n* range: 8–51; Figure 1) suggests that chromosomal fusion and fission processes, in combination with ecological adaptation, could have promoted the rapid diversification in this group [17,22,25,30,31].

**Figure 1:**
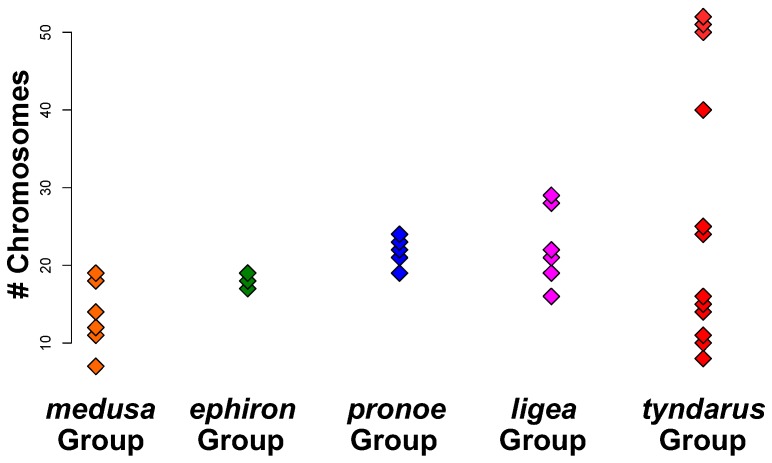
Dot plot summarizing the variation in haploid chromosome numbers among the five European *Erebia* groups according to [26]. Data is from [22]. *Erebia medusa* group (orange), *E. ephiron* group (green), *E.* pronoe group (blue), *E. ligea* group (pink), *E. tyndarus* group (red).

I tested for a phylogenetic signal of chromosomal changes in *Erebia* by combining data from a recent phylogenetic study [26] with classic karyological information on this genus (reviewed in [22]). I further aimed to identify the evolutionary mechanism that best fits the chromosomal changes. This involved testing if chromosomal changes evolved in a more neutral fashion [32], as in *Agrodiaetus* [21] or through adaptive evolution [33,34]. Given the recent radiation and the variation in chromosome numbers, particularly within in the *E. tyndarus* species complex (Figure 1), I expected that changes in chromosome numbers would be correlated with the phylogeny and hence the diversification of *Erebia*. If chromosomal changes have indeed promoted the diversification of *Erebia,* then an adaptive evolutionary model of trait evolution will likely best explain variation in chromosome numbers.

## 2. Materials and Methods 

### 2.1. Data Collection

Chromosome numbers for 31 *Erebia* species sequenced by Peña et al. [26] were available from Robinson [22]. The chromosome number for *E. graucasica* was further taken from Albre et al. [35]. In addition, I included three species for which I found chromosome numbers, and which had previously been used as outgroups (*Maniola jurtina*, *Oeneis jutta*, *Pyronia cecilia*; [26]). For each species, I selected the individuals with the most complete sequences from GenBank (Table S1). The final alignment comprised sequence data for four genes: 620 bp of the mitochondrial cytochrome oxidase subunit I (*COI*), 598 bp of the nuclear glyceraldehyde-3-phosphate dehydrogenase (*GAPDH*), 565 bp of the nuclear ribosomal protein S5 (*RpS5*) and 343 bp of the nuclear *wingless* gene (*WG*).

To increase the taxonomic scope, I searched for additional *Erebia* species whose sequences were not included in Peña et al. [26] and for which the number of chromosomes was also available [22]. Sequences of the mitochondrial *COI* gene could be retrieved for another seven species. In two cases, the available sequences were identical between two species—all of which were from the recently diversified *E. tyndarus* group, i.e., between *E. nivalis* (*n* = 11) and *E. cassioides* (*n* = 10), as well as between *E. rondoui* (*n* = 24) and *E. hispanica* (*n* = 25). For these, I retained the species with the more complete sequence data in the analysis, i.e., *E. cassioides* and *E. hispanica* (Table S1). This resulted in a dataset comprising 40 taxa, including three outgroups, sequenced for 620 bp.

### 2.2. Phylogenetic Analyses

I used PartitionFinder 2 [36] to infer the best partition scheme and associated substitution model for each codon position and gene. The resulting best partitioning scheme for the Bayesian inference is given in Table S2. For the maximum likelihood (ML)-based phylogeny, I used the GTR model with invariant sites and gamma correction (GTR+I+G) in RAxML 8.2.8. [37] with the corresponding partition scheme from PartitionFinder. I employed 1000 bootstrap replicates to assess significance. I ran RAxML for the dataset comprising either all four genes, the mitochondrial *COI* gene only or the three nuclear genes. In the latter case, data was only available for 35 taxa (Table S1). I conducted the Bayesian analysis in MrBayes 3.2.2. [38] for either dataset using, in each case, 5,000,000 generations with four chains—three heated and one cold. Trees were sampled every 1000 generations and I examined convergence and mixing based on effective sample sizes (ESS) in Tracer 1.6.0. [39]. Finally, I removed 80% of the MrBayes trees as burn-in and constructed a majority rule consensus tree.

To test for a phylogenetic signal of chromosome number onto the Bayesian phylogenies, I calculated three different indices for each dataset–Moran’s *I*, Blomberg’s κ and Pagel’s λ–using the package phylosignal [40] in R 3.3.1. [41]. Moran's *I* is a statistical measure for the autocorrelation of trait values on a phylogeny based on phylogenetic distances. A significant Moran’s *I* indicates that closely related species are more similar than expected by chance [42]. I calculated Moran’s *I* both across the entire phylogeny as well as locally between tips. While Moran’s *I* makes no assumptions about the underlying evolutionary model, both κ and λ assume a Brownian Motion (BM) model of neutral trait evolution ([43], see also below). Deviations from expectations under BM may thus also reflect that the underlying phylogeny could follow a different evolutionary mechanism as for example captured with the Ornstein-Uhlenbeck model ([42], see below). Blomberg’s κ measures the strength of a phylogenetic signal as the ratio of the mean squared error of the tip data in relation to the mean squared error of the phylogenetic (co)variance matrix. Under BM, κ is expected to be 1. κ > 1 suggests that close relatives on the tree are more similar than expected under BM, whereas κ < 1 indicates that closely related species resemble each other less than expected under BM [42,43]. Finally, Pagel’s λ is a phylogenetic scaling parameter and measures the phylogenetic dependence of a trait. λ ranges from 0—where the studied trait evolves independently of the phylogeny—to 1—where the studied trait evolves under BM [42]. I tested each index against the null hypothesis of absence of a phylogenetic signal in which case trait values would be randomly distributed along the phylogeny, using 1000 randomization steps with phylosignal. To also account for phylogenetic uncertainty, I calculated all indices for each of the 1000 post burn-in Bayesian phylograms using the package sensiPhy [44].

I compared the fit between the number of chromosomes with the phylogeny using three different evolutionary models implemented in the package mvMORPH [45]: (i) BM, which is based on a random walk process [32]. (ii) Ornstein-Uhlenbeck (OU), which fits a random walk with a central tendency towards a particular range of phenotypes representing an adaptive optimum [34]. (iii) Early Burst (EB), which assumes initially rapid evolution that is followed by relative stasis [33]. While BM is a model of neutral evolution, both OU and EB assume adaptive evolutionary mechanisms. I fitted each model to all of the 1000 post burn-in Bayesian phylograms and compared them using Aikaike’s information criterion corrected for finite sample sizes (AICc). I performed all statistical analyses in R. The sequence alignments and estimated trees are available from the Zenodo repository (10.5281/zenodo.1183375).

## 3. Results

### 3.1. Phylogenetic Reconstruction

**Figure 2:**
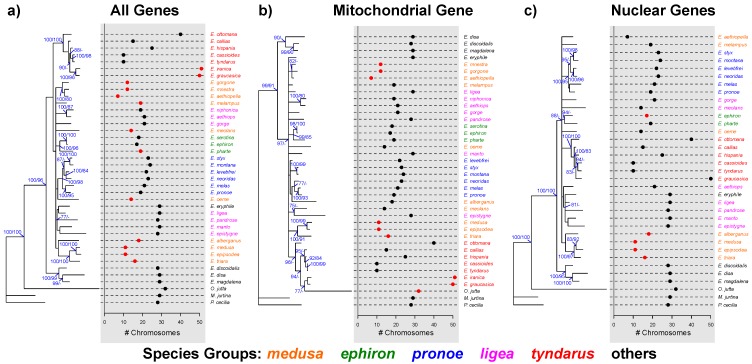
Majority rule consensus phylograms, each based on 1000 post burn-in trees using either (**a**) all four genes combined (*n* = 40 taxa), (**b**) the mitochondrial *COI* gene (*n* = 40 taxa), and (**c**) sequences of three nuclear genes (glyceraldehyde-3-phosphate dehydrogenase (*GAPDH)*, ribosomal protein S5 (*RpS5)*, wingless (*WG)*) combined (*n* = 35 taxa). Numbers at each node indicate the posterior Bayesian probabilities followed by the bootstrap support for maximum likelihood trees. Only support values ≥75% are indicated. The haploid chromosome number is indicated for each species. Red dots highlight cases with a significant local Moran’s *I* based on 1000 permutations. Species label colors designate different *Erebia* groups (see also Figure 1).

All ESS values for the 1000 post burn-in trees from the MrBayes analyses were >200, suggesting an adequate sampling of the posteriors. Branch support generally was generally in agreement between the Bayesian and ML inferences (Figure 2), where phylogenetic resolution was highest when all four genes were analyzed together (Figure 2a). This was further highlighted by the position of the outgroup species *O. jutta*, which occurred outside the *Erebia* clade with 100% support when using all four genes combined (Figure 2a) or the three nuclear genes (Figure 2c) but was nested among *Erebia* species when only the mitochondrial *COI* gene was used (Figure 2b), suggesting little differentiation at this marker. Independent of the analyzed dataset, species from the European *E. ephiron*, *E. pronoe*, and *E. tyndarus* groups form distinct clades, whereas the *E. medusa* and *E. ligea* groups are not clearly resolved (Figure 2).

### 3.2. Phylogenetic Signals

Local estimates of Moran’s *I* across the majority rule consensus trees suggest a consistently significant correlation between chromosomal numbers and the respective phylogeny among all species of the *E. medusa* group when analyzing all genes combined (Figure 2a). Using only mitochondrial or nuclear genes, this was, however, only true for six and four species of the *E. medusa* group respectively (Figure 2b,c). Significant local Moran’s *I* further occurs in two species of the *E. tyndarus* group (*E. iranica* and *E. graucasica*) when analyzing all genes combined and the mitochondrial *COI* gene only (Figure 2a,b).

**Figure 3:**
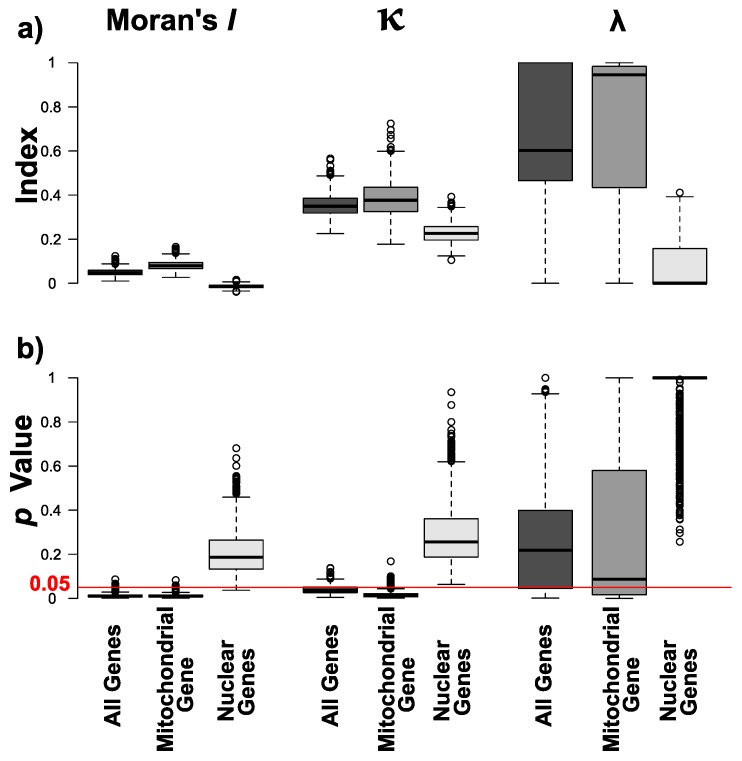
Summary of phylogenetic estimates across 1000 post burn-in trees for each dataset. Boxplots depict (**a**) the observed estimates for Moran’s *I*, Blomberg’s κ and Pagel’s λ with their (**b**) associated *p* values. The red line highlights a *p* value cut-off of 0.05.

Using the majority rule consensus phylograms, I found a small but significant degree of overall autocorrelation (Moran’s *I*: 0.040, *p* = 0.003), where κ (κ: 0.336, *p* = 0.030) but not λ (λ: 0.245, *p* = 0.500) were significant for the dataset comprising all four genes. Phylogenetic signals were stronger for the mitochondrial (*COI*) dataset (Moran’s *I*: 0.074, *p* = 0.008; κ: 0.439, *p* = 0.006; λ: 0.601, *p* = 0.327), but absent when only nuclear genes were used (Moran’s *I*: 0.019, *p* = 0.224; κ: 0.238, *p* = 0.258; λ: 0.472, *p* = 0.261). When looking at the phylogenetic uncertainty across the 1000 post burn-in phylograms, I found a generally significant (i.e., *p* < 0.05) overall autocorrelation—reflected by Moran’s *I*—and κ, whereas λ varied for the two datasets that comprised the mitochondrial *COI* gene. The phylogenetic signals for *I* and κ were generally stronger when only *COI* was used. For the dataset comprising only nuclear genes, significant estimates were mostly absent across the 1000 post burn-in phylograms (Figure 3).

**Figure 4:**
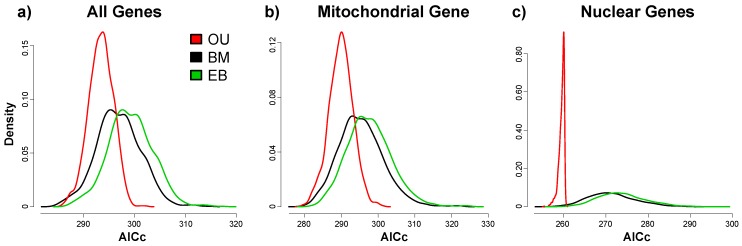
Density distributions for Akaike’s information criterion, corrected for finite sample sizes (AICc) estimated for three different models across the 1000 post burn-in trees for each dataset: black —Brownian motion (BM), red—Ornstein-Uhlenbeck (OU), green—early burst (EB).

Independent of the analyzed dataset, the OU models significantly outperformed both the EB (all genes: Δ_AICc_ = 5.82, *t*_1,999_ = 78.7, *p* < 0.001; mitochondrial gene: Δ_AICc_ = 7.77, *t*_1,999_ = 73.6, *p* < 0.001; nuclear genes: Δ_AICc_ =14.30, *t*_1,999_ = 87.6, *p* < 0.001) and BM (all genes: Δ_AICc_ = 3.48, *t*_1,999_ = 47.1, *p* < 0.001; mitochondrial gene: Δ_AICc_ = 5.44, *t*_1,999_ = 51.6, *p* < 0.001; nuclear genes: Δ_AICc_ = 11.90, *t*_1,999_ = 72.9, *p* < 0.001) models based on paired *t*-tests between their respective AICc values (Figure 4).

## 4. Discussion

Several classic models of speciation predict that chromosomal changes, such as inversions or the fusion and fission of entire chromosomes, can advance the speciation process by acting as intrinsic barriers to gene flow [12,46]. This view has been challenged by theoretical and empirical advances, which suggest that gene flow can occur despite such genomic rearrangements [13,18]. The potential for chromosomal speciation by combining genetic and karyotypic data has, however, only been assessed for a restricted set of taxa [13,20,21,47]. Combining existing sequence data with karyotypic information, I tested for a phylogenetic signal underlying the varying chromosome numbers in *Erebia* butterflies (Figure 2 and Figure 3). I also inferred the underlying evolutionary mechanisms by comparing different models of trait evolution (Figure 4). I found a small yet significant level of overall correlation between chromosome numbers and the phylogenetic inference as measured by Moran’s *I*, when the mitochondrial *COI* gene was included. Closely related species were therefore more similar than expected by chance. This is likely driven by groups of *Erebia*, particularly *E. medusa,* that show consistently significant local correlations (*I*) among datasets (Figure 2), as well as conservatism in terms of their chromosome numbers (Figure 1). The absence of such a correlation when only nuclear genes were used may further implicate different evolutionary histories between the nuclear and the mitochondrial genome [26]. Mitochondrial and nuclear discordance is a widespread phenomenon in animals (reviewed in [48]). This is often a result of incomplete lineage sorting and/or introgression in the nuclear genome [48], the latter being common in tropical *Heliconius* butterflies [49]. The endosymbiotic bacterium *Wolbachia* can cause similar discordance in butterflies [50,51]. Introgression and hybridization also occurs in *Erebia* [17,29], yet broader taxonomic and genomic studies are needed to determine the extent and causes of cytonuclear discordance observed in this study.

For the datasets comprising the mitochondrial *COI* gene, Blomberg’s κ was significantly smaller than one (Figure 3). Under a Brownian motion model of trait evolution, this suggests that closely related species are less similar than expected [43], e.g., when chromosomal changes occur independently from each other during allopatric phases [21]. Alternatively, the observed reduction in κ could be caused by adaptive trait evolution. This is suggested by the better fit of the Ornstein-Uhlenbeck model of trait evolution compared to the neutral model [42]. However, the restricted taxonomic sampling and the limited amount of available genetic data combined with the inconclusive results when estimating Pagel’s λ (Figure 3), the placement of *O. jutta* within the *Erebia* clade (Figure 2b) and the abundance of polytomies highlight the limits of this study (Figure 2). Whereas Moran’s *I* has been shown to be a robust estimator of phylogenetic autocorrelation in the presence of polytomies [42], phylogenetic uncertainty can inflate Blomberg’s κ under a Brownian motion model [52]. That said, the fact that the phylogenies comprising *COI* are better resolved indicates that the relative difference in κ between datasets (Figure 3) is unlikely to have been caused by such an artifact.

The Ornstein-Uhlenbeck model of adaptive trait evolution significantly outperformed the neutral Brownian motion model for all three genetic datasets (Figure 4). Such differences can also emerge by chance, i.e., when differences in likelihood estimates between two models are small (i.e., Δ_AICc_ < 2 [53]). This is, however, not the case for *Erebia* (Figure 4). The better fit of the Ornstein-Uhlenbeck model contrasts with a recent study on *Agrodiaetus* butterflies, where chromosomal changes were best explained by neutral Brownian motion [21]. Differences in the evolutionary history between the two butterfly groups may account for this inconsistency. *Agrodiaetus* butterflies are thought to have mainly diversified in allopatry, where they gradually accumulate karyotypic changes [23,47,54]. This often results in small chromosomal differences between closely related species [21]. Upon secondary contact between closely related species, *Agrodiaetus* butterflies evolve increased phenotypic differentiation through reinforcement, which subsequently promotes coexistence [23,47,54]. In contrast, patterns of chromosomal evolution differ among distinct *Erebia* groups. Both the *E. medusa* and *E. ligea* group show conservatism in terms of chromosome numbers compared to the *E. tyndarus* group (Figure 1). *Erebia* species from the same group do, moreover, coexist while often being phenotypically cryptic, but show ecological differentiation in their microhabitat use [31]. This can be further reinforced in contact zones of closely related species, as in the *E. tyndarus* group [25,30,31]. The signal of adaptive evolution (Figure 4) observed in *Erebia* could therefore have been driven by (i) stabilizing selection in *E. medusa* and *E. ligea* [55] and/or (ii) the rapid diversification of the *E. tyndarus* group. A resolved phylogeny with broader taxonomic sampling is required to distinguish between these scenarios. Whether the chromosomal changes initiated divergence in this group also cannot be determined with the current dataset.

Taken together, the adaptive phylogenetic responses observed here (Figure 4), together with the recent radiation of the *E. tyndarus* group, demonstrate the utility of the *Erebia* genus to study the role of chromosomal fusion and fission during speciation. Further studies using genome-wide data are now needed to overcome potential issues associated with the observed phylogenetic uncertainties caused by polytomies [56] and to assess the role of interspecific gene flow in relation to incomplete lineage sorting. Genome-wide data would also allow the inclusion of even more recently diverged species, which were excluded from this study because they had identical *COI* sequences. Particularly *E. nivalis*, which has a different chromosome number (*n* = 11) than its two close relatives *E. cassioides* and *E. tyndarus* (both *n* = 10) may provide a great system to study the role of recent chromosomal changes during speciation as species ranges overlap and interspecific gene flow is still possible [17,29]. Lastly, because similar variation in chromosome numbers occurs in several other Lepidoptera groups [22,57], the study of *Erebia* may help to shed light on how and why chromosome numbers vary within this highly diverse order.

## Supplementary Materials

The following are available online at http://www.mdpi.com/2073-4425/9/3/166/s1. Table S1: Accession numbers for each gene and individual included in this study, Table S2: Codon partitioning scheme for each gene used for the Bayesian analysis.
